# Supplementary material for: S2 Peptide-Conjugated SARS-CoV-2 Virus-like Particles Provide Broad Protection against SARS-CoV-2 Variants of Concern
Source: Vaccines (Basel). 2024 Jun 18;12(6):676. doi: 10.3390/vaccines12060676 (PMC11209314; doi:10.3390/vaccines12060676)
Supplement: Supplementary file 1 [file vaccines-12-00676-s001.zip › vaccines-3037108-supplementary.pdf]

Supplementary Materials

S2 Peptide-Conjugated SARS-CoV-2 Virus-Like Particles Provide Broad Protection against SARS-CoV-2 Variants of Concern

Chang-Kyu Heo [1], Won-Hee Lim [1,2], Ki-Beom Moon [3], Jihyun Yang [4], Sang Jick Kim [5], Hyun-Soon Kim [3], Doo-Jin Kim [6] and Eun-Wie Cho [1,2,*]

1 Rare Disease Research Center, Korea Research Institute of Bioscience and Biotechnology (KRIBB), 125 Gwahak-ro, Youseong-gu, Daejeon 34141, Republic of Korea; ckheo40@kribb.re.kr (C.K.H.); whlim4028@gmail.com (W.H.L); ewcho@kribb.re.kr (E.W.C.)
2 Department of Functional Genomics, KRIBB School of Bioscience, Korea University of Science and Technology, Daejeon, Republic of Korea; whlim4028@gmail.com (W.H.L); ewcho@kribb.re.kr (E.W.C.)
3 Plant Systems Engineering Research Center, KRIBB, Daejeon, Republic of Korea; irony83@kribb.re.kr (K.B.M.); hyuns@kribb.re.kr (H.S.K.)
4 Infectious Disease Research Center, KRIBB, Daejeon, Republic of Korea; jhyang@kribb.re.kr (J.Y.)
5 Synthetic Biology and Bioengineering Research Center, KRIBB, Daejeon, Republic of Korea; sjick@kribb.re.kr (S.J.K.)
6 College of Medicine, Chungbuk National University, Cheongju, Chungcheongbuk-do, Republic of Korea; golddj@cbnu.ac.kr (D.J.K.)
* Correspondence: ewcho@kribb.re.kr; Tel.: +82-42-860-4155

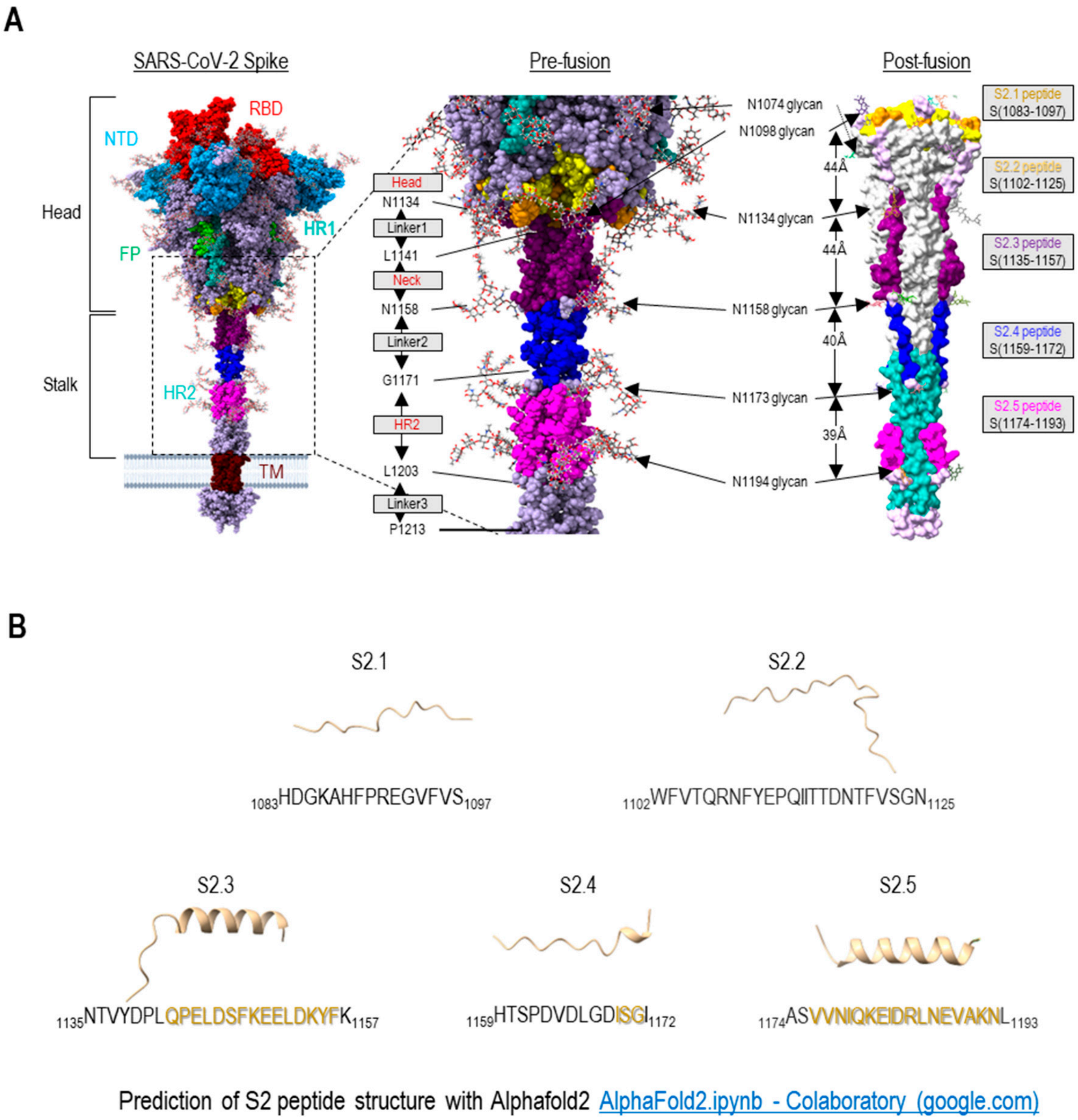

Figure S1. Structural features of the SARS-CoV-2 S2 antigenic peptides used in this study. **(A)** Modeled structure of the full-length glycosylated spike in its pre- and postfusion states as determined by Cryo-EM (PDB ID 6VSB and 6XRA, respectively). The head, linker, and neck domains in the prefusion state structure were annotated based on a previous study [25]. In the postfusion state, five N-glycans were arranged on one side of the 6-helical bundle structure, with approximately 40 Å of space between these glycans. Five peptide sequences corresponding to the regions between N-glycans (S2.1, S2.2, S2.3, S2.4, and S2.5) were selected as antigenic epitope candidates, which are depicted in distinct colors. **(B)** Prediction of the S2 peptide structure was performed using AlphaFold2 protein structure prediction software. The structure of each S2 peptide, corresponding to the sequences provided, was predicted using Alphafold2 (AlphaFold2.ipynb-Colaboratory (google.com)), and molecular graphics were visualized using the molecular visualization program UCSF ChimeraX. The helical structures of each peptide are highlighted using bold brown characters.

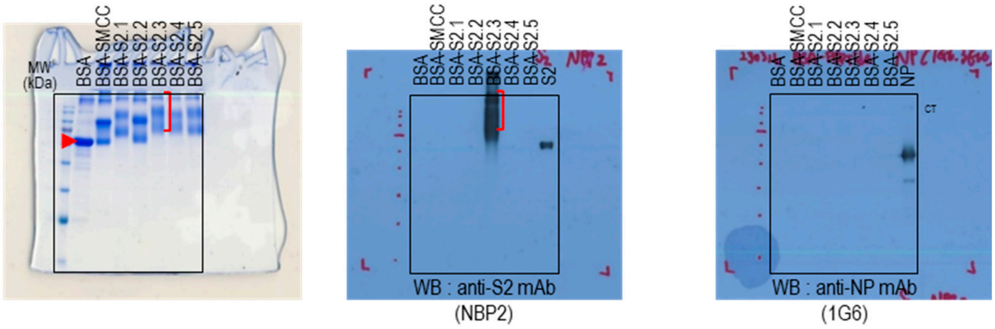

Figure S2. The original images of SDS-PAGE and western blots of Figure 1B.

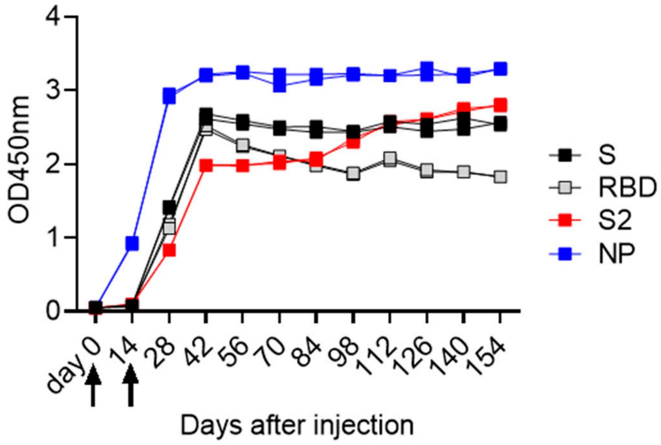

Figure S3. Total IgG response in sera from mice immunized with inactivated SARS-CoV-2 (IAV). Antisera from immunized mice (n = 3) were collected at specified time points after immunization. Antibody responses against recombinant SARS-CoV-2 antigens, including S, S2, NP, and RBD, were examined. Each dot represents the measurement of an individual serum, and the graph displays the mean ± standard deviation (SD) of replicate wells.

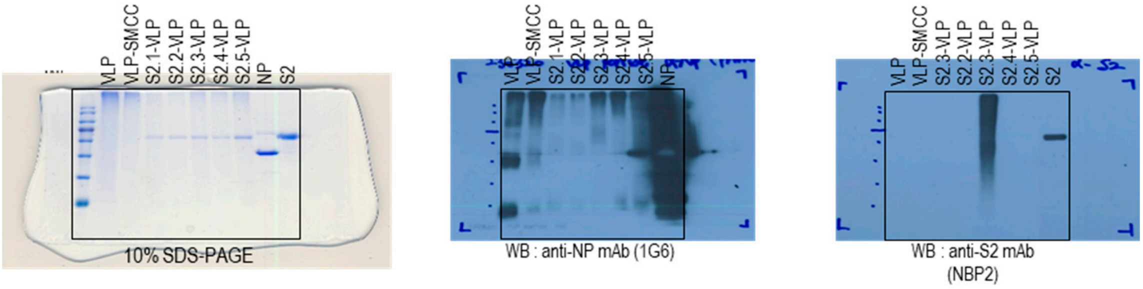

Figure S4. The original images of SDS-PAGE and western blots of Figure 2B.

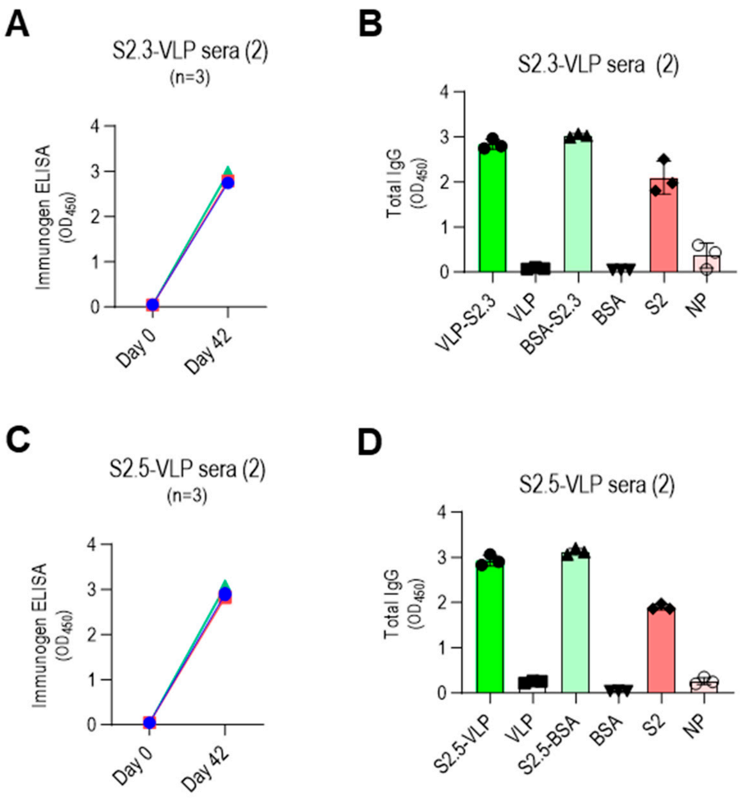

Figure S5. Specific total IgG responses to immunization with S2.3- or S2.5-conjugated VLP. After confirming the immune responses to S2-VLPs (Figure 2F), additional immunization with S2.3- or S2.5-VLPs was performed following the protocol presented in Figure 2E. After the second boost, sera were collected, and ELISA was performed for immunogens (A, C) or SARS-CoV-2 antigens (B, D). Each dot represents the antibody response of individual mice to S2 peptide-VLP immunization and displays the mean ± SD of replicate wells.

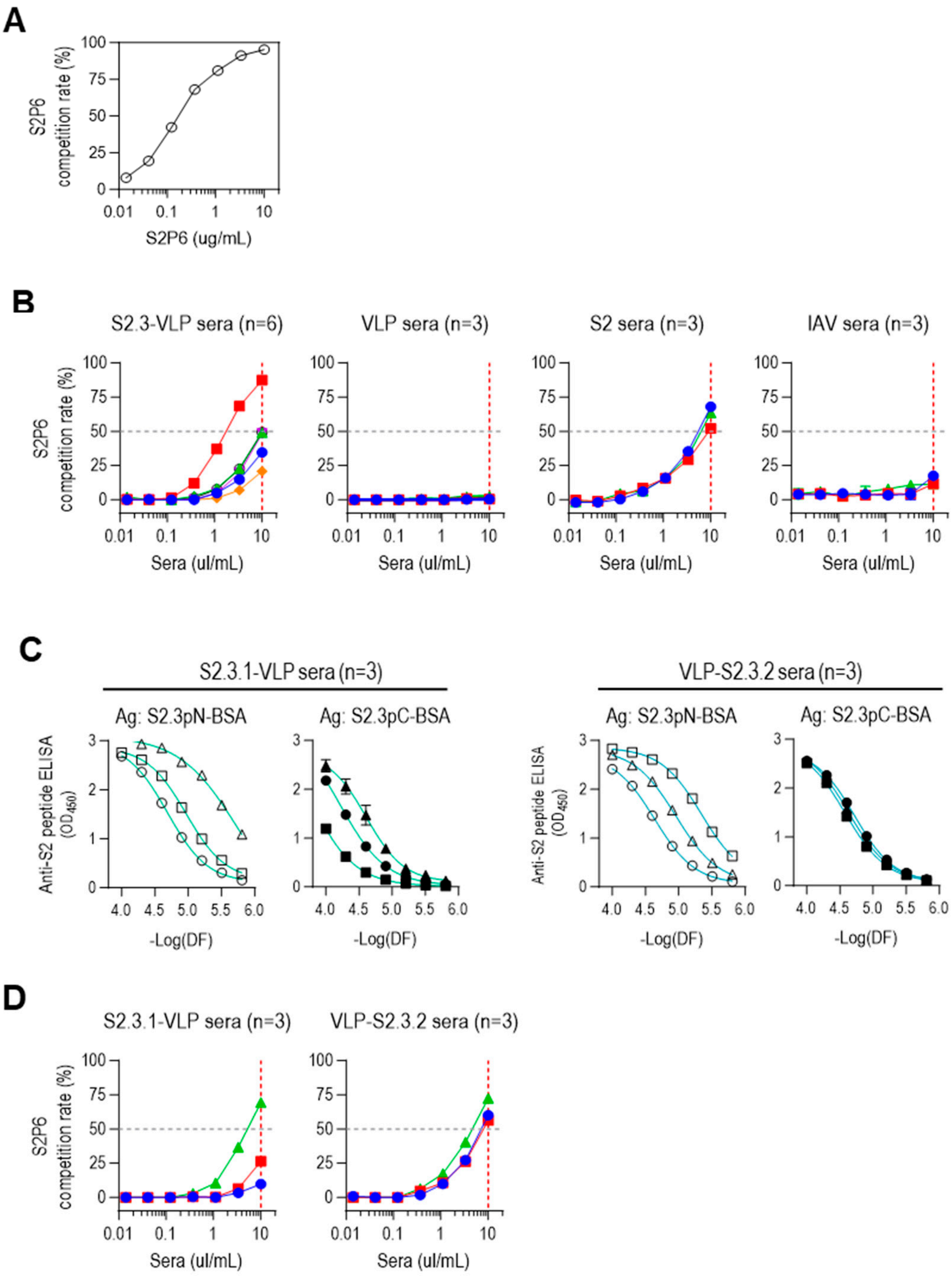

Figure S6. S2P6-like activity of S2.3 peptide-conjugated VLP-immunized sera. **(A)** Standard reaction of a competitive ELISA for quantifying S2P6-like activity. The recombinant S2 antigen was coated on a MaxiSorp plate. Serially diluted S2P6 antibody solutions were added to the well along with 10 ng HRP-conjugated S2P6. After incubation, S2P6-HRP activity was measured, and the S2P6 competition rate (%) was determined. **(B)** S2P6-like activity of immunized sera. Each plot represents the measurement of an individual mouse (n = 6 or 3) and displays the mean ± SD of replicate wells. **(C)** S2.3.1-VLP or VLP-S2.3.2 were immunized following the immunization regimen (n = 3/each immunogen), as shown in Figure 2E. The reactivity of antisera after the second boost against S2.3pN or S2.3pC epitopes was analyzed using serial dilutions. **(D)** S2P6-like activity of S2.3.1-VLP or VLP-S2.3.2-immunized sera. Each plot represents the measurement of an individual mouse and displays the mean ± SD of replicate wells.

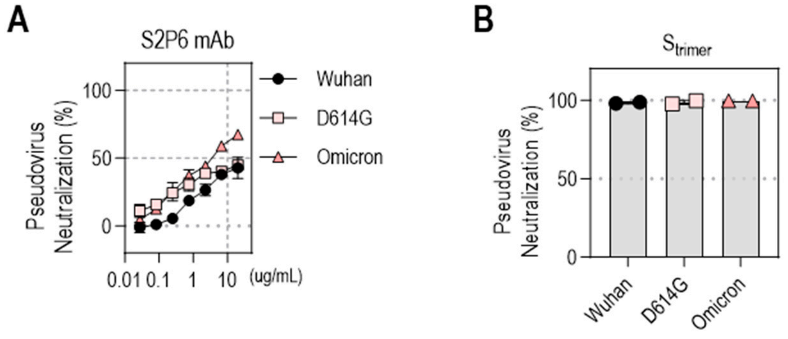

Figure S7. SARS-CoV-2 variant-neutralization potential of antisera against S-related antigens. **(A)** The neutralization potential of a broadly neutralizing S2-specific monoclonal antibody S2P6 was assessed against the pseudotyped SARS-CoV-2 prototype (Wuhan) or variants (D614G and Omicron BA2). S2P6 showed a pseudovirus neutralization potential of approximately 50% at a concentration of 20 μg/mL without significant differences in virus variant types. **(B)** The neutralization potential of antisera from mice immunized with S_{trimer} (*n* = 2). The neutralization potential was evaluated using a pseudotyped SARS-CoV-2 prototype (Wuhan) or variants (D614G and Omicron BA2) and 20 μL of serum collected on day 42 after immunization, with triplicate measurements for each sample. Each symbol displays the neutralization potential of each serum, and the values represent the mean ± SD. The luminescence value of the pseudovirus infection without sera was used as the negative control.
